# Genomic architecture of migration timing in a long-distance migratory songbird

**DOI:** 10.1038/s41598-023-29470-7

**Published:** 2023-02-10

**Authors:** Evelien de Greef, Alexander Suh, Matt J. Thorstensen, Kira E. Delmore, Kevin C. Fraser

**Affiliations:** 1 Department of Biological Sciences, University of Manitoba, Winnipeg, R3T 2N2 Canada; 2 Department of Organismal Biology, Uppsala University, 752 36 Uppsala, Sweden; 3 School of Biological Sciences, University of East Anglia, Norwich, NR4 7TU UK; 4 Department of Biology, Texas A&M University, College Station, TX 77843 USA

**Keywords:** Ecology, Genetics

## Abstract

The impact of climate change on spring phenology poses risks to migratory birds, as migration timing is controlled predominantly by endogenous mechanisms. Despite recent advances in our understanding of the underlying genetic basis of migration timing, the ways that migration timing phenotypes in wild individuals may map to specific genomic regions requires further investigation. We examined the genetic architecture of migration timing in a long-distance migratory songbird (purple martin, *Progne subis subis*) by integrating genomic data with an extensive dataset of direct migratory tracks. A moderate to large amount of variance in spring migration arrival timing was explained by genomics (proportion of phenotypic variation explained by genomics = 0.74; polygenic score *R*^2^ = 0.24). On chromosome 1, a region that was differentiated between migration timing phenotypes contained genes that could facilitate nocturnal flights and act as epigenetic modifiers. Overall, these results advance our understanding of the genomic underpinnings of migration timing.

## Introduction

Climate change affects spring phenology in temperate zones and could have significant, negative impacts on migratory animals^1^. For example, migrants must synchronize arrival at breeding grounds to coincide with seasonal resources availability^2^. These resources are becoming available earlier and it is unclear if migrants will be able to match these advances in timing, potentially leading to substantial population declines^3^. Migration phenology may be influenced by many factors, including extrinsic factors such as rainfall^4^ and wintering habitat conditions^5^. However, migration timing is thought to be largely endogenously controlled^6^, and thus knowledge of its genetic architecture (e.g., the identity, number, and location of genetic loci involved) is essential for predicting how migrants will respond to phenological changes that accompany climate change.

Previous genetic studies provide important insights regarding migration timing, such as in genes associated with circadian and circannual rhythms^7–12^; however, results vary across species^13^ and are limited to small portions of the genome. Another limitation associated with earlier studies was an inability to quantify migratory behavior in the wild—prior to 2007, it was not possible to track animals < 100 g on migration^14^ and most migratory avian species fall into this size class. We overcame these limitations here, combining high resolution genomic data with an extensive migration tracking dataset for purple martins (*Progne subis subis*), while specifically focusing on spring arrival timing given the importance of matching timing with resources critical for breeding. The purple martin is a Nearctic-neotropical migrant that travels over 7000 km between North America and South America^15^ and exhibits extensive latitudinal variation in migration timing. It is thus a powerful system to study migration genomics. For example, individuals breeding in the southern edge of the range in Florida may arrive as early as mid-January, while their northern counterparts in Alberta may arrive as late as June^16^.

Our objective was to examine the genomic architecture of migration timing by assembling a reference genome for the purple martin and integrating sequencing data with light-level geolocator tracks. We examined results from genome-wide association studies (GWAS), to calculate the proportion of phenotype variation explained by our genomic data (PVE) along with presence of any associated single nucleotide polymorphisms (SNPs). We also calculated polygenic scores (PGS) to assess the genetic predisposition of migratory timing. Lastly, we conducted a genomic differentiation analyses between different migratory phenotypes to identify regions in the genome that may influence this trait. This study expands our understanding of the whole-genome contribution to migration and yields insight into migration timing behavior.

## Results

### Reference and resequencing data

The final *P. subis* reference genome assembly based on long reads and linked reads was 1.17 Gb in length, consisted of 2896 scaffolds, had an N50 scaffold length of 6.13 Mb and an N50 contig length of 3.08 Mb. The annotation included 12,686 genes (SI Appendix, Table S1). The assembly length was similar to other avian genomes, which are typically between 1.0 and 1.2 Gb^17^. BUSCO analysis revealed that the *P. subis* genome was relatively complete with 91% of avian orthologs detected as complete sequences (89.1% being single-copy and 1.9% being duplicated), which was in the range of other non-model avian genomes^18^. We aligned resequencing data for 87 individuals to this reference resulting in 4.6 million SNPs after filtering. All these individuals were tracked on migration with light-level geolocators yielding precise estimates for migratory timing.

### Genomic architecture of migration timing

**Figure 1:**
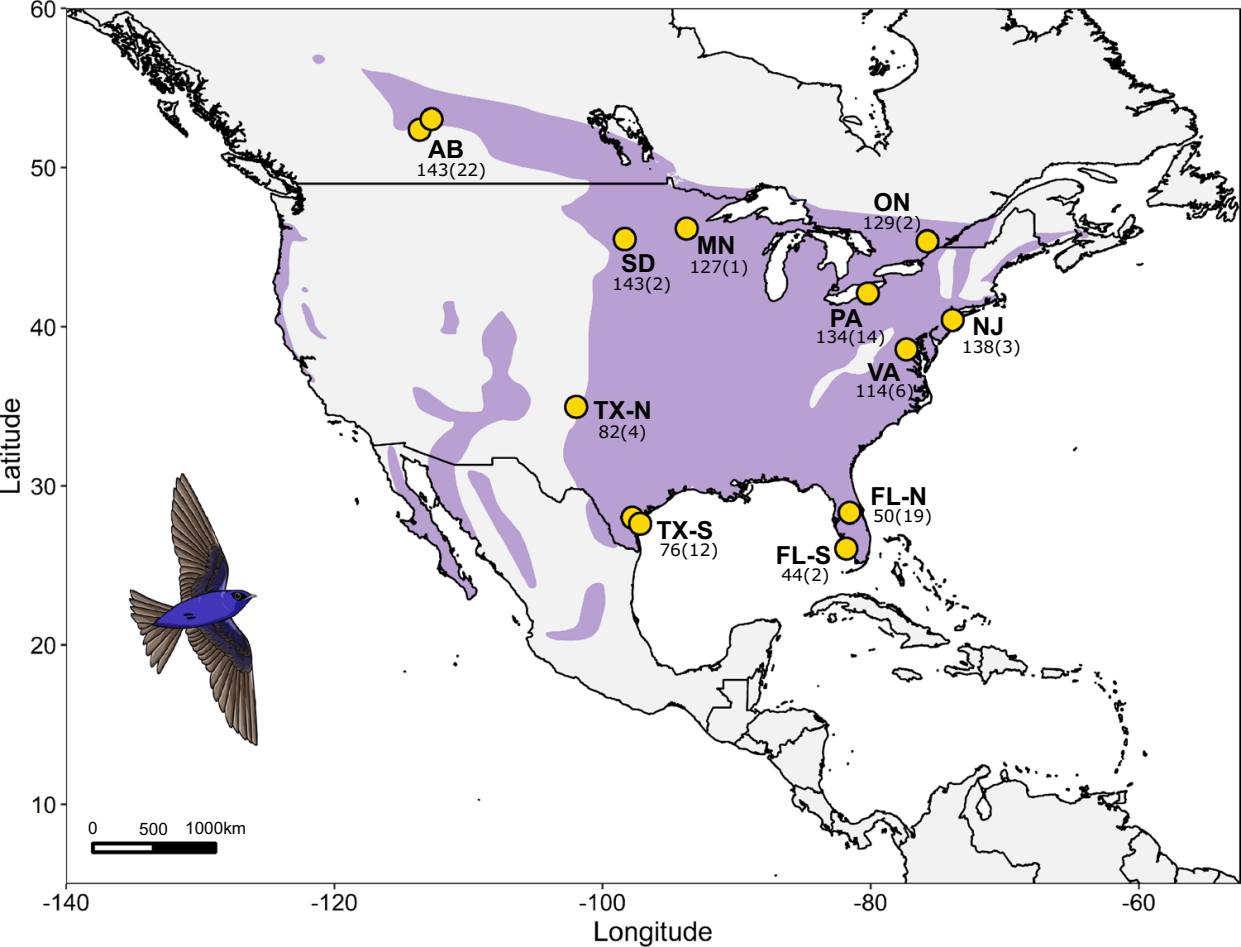
Purple martin breeding distribution (purple), including sampling sites for 87 individuals in their North American breeding range (yellow). The mean arrival date for each site is in ordinal date format (day of year), with the site sample size provided in parentheses. The purple martin range map layer was obtained through the IUCN red list database.

Birds in this study exhibited considerable latitudinal variation in migratory timing (sampling locations in Table S2), ranging over 131 days for spring arrival dates (Fig. S1). Sampling locations in the breeding grounds are displayed in Fig. 1.

**Figure 2:**
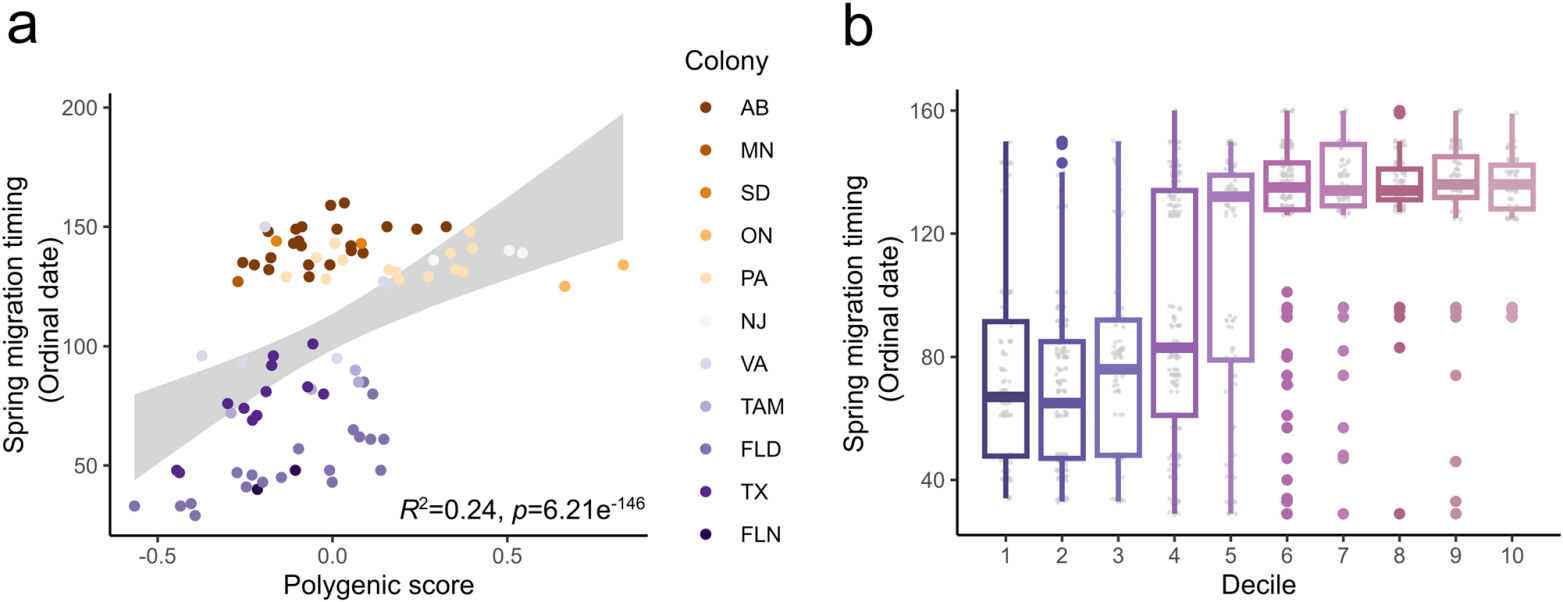
(**a**) Polygenic scores of spring migration timing for purple martins (*n* = 87) colored in order by latitude, and linear regression 95% confidence interval is colored in gray. (**b**) Predicted polygenic scores (PGS) partitioned by decile (lowest PGS = decile 1, highest PGS = decile 10), showing individuals (raw points in gray dots and results summarized in box plots) in lowest deciles had earlier migration timing compared with individuals in higher deciles with later timing.

Estimates of PVE from Bayesian sparse linear mixed models (BSLMMs)^19^ were high, with a median value of 0.74 and 89% equal-tailed interval (ETI) of 0.10–1.00. PGS estimated from linear mixed models^20^ were strongly correlated with spring migration timing (*R*^2^ of 0.24*, p* < 0.001, Fig. 2a). We assessed predictive power of the PGS model with 100 jackknife cross-validation partitions, and found that birds with lower PGS deciles exhibited earlier spring migration timing compared with individuals in higher PGS deciles with later spring migration timing (Fig. 2b).

**Figure 3:**
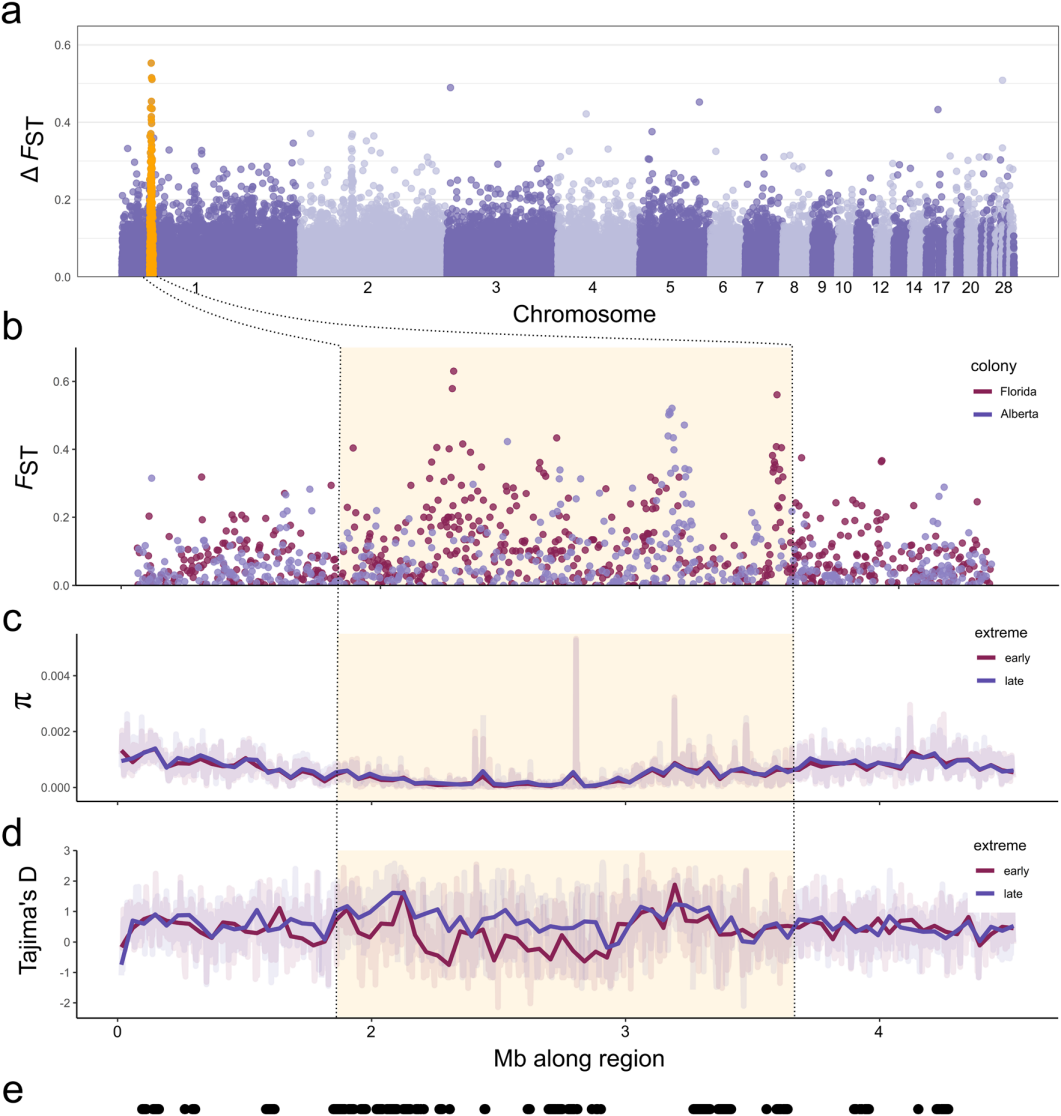
(**a**) Net genetic differentiation (Δ*F*_ST_) across autosomes in 5 kb non-overlapping windows between earliest and latest spring migrants. The elevated region on chromosome 1 (defined as scaffolds with homology to chicken chromosome 1) is highlighted in orange, with plots examining this region to show (**b**) *F*_ST_ between early and late migrants within Alberta and within Florida, (**c**) nucleotide diversity (π) between earliest and latest migrants, (**d**) Tajima’s D between earliest and late migrants, and (**e**) location of genes in this region as black dots.

BSLMMs did not identify any specific genomic regions linked to migratory timing (Fig. S2)*.* However, a survey of net genomic differentiation (Δ*F*_ST_) between the earliest and latest spring migrants in our dataset did reveal a region of elevated differentiation on chromosome 1 (Fig. 3). Δ*F*_ST_ controls for processes unrelated to migration that could elevate *F*_ST_ (including population structure, see “Methods”). This elevation was additionally present in comparisons of early and late migrants within populations in Florida (southernmost colony) and Alberta (northernmost colony) (Fig. 3b) suggesting population structure did not generate this pattern. Reductions in nucleotide diversity and Tajima’s D indicative of a selective sweep are also present in this region, which covers 2 Mb region and consist of 13 genes (Table S3) including *ppfia2* and *nts*, which may be related to sleep^21,22^, and *mettl25* and *acss3* that may serve as epigenetic modifiers by adding methyl groups to DNA^23^ or remodeling chromatin and regulating gene expression^24^.

## Discussion

We used one of the largest tracking datasets available for a long-distance migratory songbird and a genome-wide SNP dataset to reveal the genetic underpinnings of migration timing. The results demonstrate a genetic basis to migration timing in the purple martin. A previously unidentified 2 Mb genetically differentiated region was found on chromosome 1, illuminating a candidate genomic region underlying migration timing.

Phenotypes that vary across individuals are a result of both environmental and genetic factors, and PVE represents the proportion of variance attributed to genetic factors. The large PVE estimate (0.74) is evidence that variation in migration timing is largely influenced by genetics. The predictive utility of the polygenic model across multiple deciles showed it is possible to distinguish early and late migrants using genetic variants, which could potentially aid in estimating a birds’ phenotype in the wild. With overlapping wintering grounds among purple martin colonies^15^, the model may also help predict an individuals’ timing tendency in addition to breeding region when captured during the winter. Some species demonstrate phenotypic plasticity in migration timing^25^, including purple martins^26^. While the high genomic variation explaining migratory timing does not preclude phenotypic plasticity, it suggests that changes in timing would also need to occur through microevolutionary processes. It is important to understand the sources of considerable genetic variation in migratory traits^6^, and future work will inform how influences such as standing genetic variation (presence of more than one allele at a locus in a population) may play a role in facilitating rapid microevolutionary changes^27^. While our sample size limits our estimates of PVE (the ETIs were quite wide), our PGS analysis supported the potential to predict individual migration timing with genomic data. Given the present sample size, it is remarkable that a substantial proportion of variation was explained with genomics. Whether this proportion could be even greater with larger sample sizes^28,29^ could be determined in future studies. However, collecting enough samples to capture strong statistical power in tests of genomic associations with phenotypes in field-based wildlife research will be challenging.

The lack of genomic loci in the GWAS significantly associated with spring migration timing suggests this trait is controlled by many alleles of small effect, which may have been undetected, or could have been located in assembly gaps^17^. However, elevated genomic differentiation on chromosome 1 provides evidence for a potential connection between migration timing in purple martins with some genes related to rest and others that could act as epigenetic modifiers. *Ppfia2* and *nts* are 2 of the 13 genes located in this region. *Ppfia2* has been linked to sleep and wakefulness in white-crowned sparrows^21^, and *nts* has been linked to sleep regulation in European mice^22^. While purple martins are primarily diurnal migrants, they can incorporate both day and night flights on spring migration^30^, and the former genes could play a role in these nocturnal flights. Epigenetic modifiers may play a role in migration timing, such as methylation on the polyglutamine repeat domain of the *clock* gene affecting phenology and breeding performance^31^. Therefore, it is interesting that *mettl25* and *acss3* are in a region of high differentiation between early and late migrants. *Mettl25* may be similar to *mettl25b* and encode a methyltransferase that represses gene expression^23^ and *acss3* produces acetyl-CoA which promotes gene expression by acetylating histones^32^. Acetyl-CoA is also important for generating, using, and storing energy^24^. While this study suggests associations with these genes, our current data cannot pinpoint how these genes are functioning with purple martin migration timing. Further work could elucidate these mechanisms and their roles in migration timing. While estimates of *F*_ST_ are often considered bottom-up comparisons, we compared extreme phenotypes while controlling for population differentiation to identify genomic regions associated with migration timing. Results from this approach were further supported by a comparison of early and late migrants within populations that recovered a similar elevated pattern of genomic differentiation (Fig. 3b).

This study presents novel findings on migration timing, facilitating an understanding of the components in the genomic architecture of migration timing in other long-distance migrants. The strong genomic variation and significant regions associated with purple martin migration timing could have important implications for adaptability in long-distance migrants. Many portions of the genome are conserved across other bird species and vertebrates^33^ and climate change continues to affect migratory animals all over the world. Therefore, these findings bring us closer to understanding a common basis for migration, which may have broad implications for a variety of organisms.

## Methods

### Reference and resequencing data

The reference genome was assembled using PacBio long and 10X linked reads generated for a female martin from Manitoba, Canada. We used FALCON^34,35^ to create the initial assembly, then polished and scaffolded the genome with ArrowGrid, Pilon, and ARKS^36–38^. The genome was annotated with MAKER^39^. We used skimSeq (low-coverage whole-genome sequencing) to generate resequencing data^40^ for an additional 87 birds (average coverage 2.7 × per sample). Missing genotypes were then imputed with Beagle^41^, using information from the reference, surrounding genotypes, linkage disequilibrium structure, and haplotype blocks^42^. These included 45 male and 42 female blood samples collected from 13 different breeding colonies across North America between 2008 and 2015 (Table S2). We filtered SNPs for quality (QUAL > 20, MQ > 20), maximum-missingness (20%), minor allele frequency (MAF > 0.05), Hardy–Weinberg equilibrium, and biallelic sites. Details on assembly, annotation, sequencing, and filtering are in supplementary information.

### Light-level geolocator analysis

Light-level geolocators were mounted during the breeding season using leg-loop backpack harnesses and retrieved through recapture in the following year. Purple martin behavior of aerial foraging and use of open habitats makes light-level geolocators ideal for capturing sunrises and sunsets with minimal shading. The timing of these twilights is used to estimate the daily locations of birds over the entire year, using the midpoint of rise-set events to determine longitudes and day length for estimating latitudes^43^. We analyzed twilight times with BAStag and GeoLight^44,45^, producing estimated daily locations to obtain migratory departure and arrival dates. Due to the correlation of departure with arrival timing for migratory journeys (Fig. S3), we used spring arrival date as the migration timing phenotype.

### **Genomic architecture of migration timing**

BSLMMs and LMMs were run using GEMMA^19^, where we included the covariates of sex, year, age, and the first principal component (PC1) from a PCA summarizing genetic variation in our dataset. We summarized results from these runs for PVE and used posterior inclusion probabilities (PIP) to identify specific SNPs with strong associations to the timing phenotypes. PIP is the probability that the SNP is associated with the phenotypic variation^46^ and following^47^ we considered SNPs with PIPs > 0.1 important. Polygenic models were created using the PLINK v1.9^48^ and following Choi et al. (2020)'s PGS pipeline^49^. Accuracy of the PGS model was assessed with jackknife cross-validation partitions using a random 85% of the dataset for the GWAS (n = 74) and the other 15% to test prediction accuracy (n = 13) over each of the 100 runs. We used VCFtools^50^ to estimate *F*_ST_ between the 10 earliest (originating from two Florida colonies) and 10 latest (originating from two Alberta colonies, one Virginia colony, and one Pennsylvania colony) spring migrants. Since this *F*_ST_ could be elevated by processes unrelated to migration, including linked background selection and population structure^51^, we controlled for these potential effects by subtracting *F*_ST_ between Alberta and Florida (representing the northernmost and southernmost breeding regions) from values estimated between extreme timing phenotypes. This approached has been used in crows^52^ and blackcaps^53^ to isolate differentiation associated with specific phenotypes. Additionally, we estimated *F*_ST_ between early and late migrants within Alberta and Florida populations separately to examine if we could recover the same signature of elevated *F*_ST_ in the same genomic region.

## Supplementary Information

Supplementary Information.

## Data Availability

Raw sequencing data is available on SRA NCBI BioProject PRJNA772931. Source code for reference genome and resequencing work is available at https://github.com/edegreef/PUMA-reference-genome and https://github.com/edegreef/PUMA-resequencing-data. Code for the polygenic analyses is available at https://github.com/BioMatt/PUMA_PGS.
